# Deep Q-Learning and Preference Based Multi-Agent System for Sustainable Agricultural Market

**DOI:** 10.3390/s21165276

**Published:** 2021-08-04

**Authors:** María E. Pérez-Pons, Ricardo S. Alonso, Oscar García, Goreti Marreiros, Juan Manuel Corchado

**Affiliations:** 1 BISITE Research Group, University of Salamanca, Edificio Multiusos I+D+i, Calle Espejo 2, 37007 Salamanca, Spain; eugenia.perez@usal.es (M.E.P.-P.); oscgar@usal.es (O.G.); corchado@usal.es (J.M.C.); 2 Air Institute, IoT Digital Innovation Hub, 37188 Salamanca, Spain; 3 GECAD—Research Group on Intelligent Engineering and Computing for Advanced Innovation and Development, Institute of Engineering, Polytechnic of Porto, 4200-072 Porto, Portugal; mgt@isep.ipp.pt; 4 Department of Electronics, Information and Communication, Faculty of Engineering, Osaka Institute of Technology, Osaka 535-8585, Japan; 5 Pusat Komputeran dan Informatik, Universiti Malaysia Kelantan, Bachok 16300, Kelantan, Malaysia

**Keywords:** multi-agent systems, decision support systems, sustainable agriculture, deep Q-learning, IoT, edge computing

## Abstract

Yearly population growth will lead to a significant increase in agricultural production in the coming years. Twenty-first century agricultural producers will be facing the challenge of achieving food security and efficiency. This must be achieved while ensuring sustainable agricultural systems and overcoming the problems posed by climate change, depletion of water resources, and the potential for increased erosion and loss of productivity due to extreme weather conditions. Those environmental consequences will directly affect the price setting process. In view of the price oscillations and the lack of transparent information for buyers, a multi-agent system (MAS) is presented in this article. It supports the making of decisions in the purchase of sustainable agricultural products. The proposed MAS consists of a system that supports decision-making when choosing a supplier on the basis of certain preference-based parameters aimed at measuring the sustainability of a supplier and a deep Q-learning agent for agricultural future market price forecast. Therefore, different agri-environmental indicators (AEIs) have been considered, as well as the use of edge computing technologies to reduce costs of data transfer to the cloud. The presented MAS combines price setting optimizations and user preferences in regards to accessing, filtering, and integrating information. The agents filter and fuse information relevant to a user according to supplier attributes and a dynamic environment. The results presented in this paper allow a user to choose the supplier that best suits their preferences as well as to gain insight on agricultural future markets price oscillations through a deep Q-learning agent.

## 1. Introduction

Environmental factors such as the consequences of climate change [1] directly affect producers in the agricultural price setting process [2]. Agricultural trade is expected to grow at roughly half the rate of the past ten years [3]. Nevertheless, for most commodities, the share of total production that is being traded on global markets will remain relatively constant. Technology will also be more important in guaranteeing global food security, due to natural resource constraints in many countries [3]. In this regard, the concept of bioeconomy is gaining importance within the European Union objectives for the 2030 Agenda and its Sustainable Development Goals, and new indicators to measure the performance of bioeconomy sectors are emerging [4]. Globalization and the possibility of exporting and importing, as well as competing on a larger market, have led to a paradigm in which agricultural products are a financial indicator of the economy and take part in the prices of future markets. Agricultural commodity prices also tend to correlate with trends on energy markets, with oil prices experiencing unusual rises and falls in recent years [5]. Although agricultural inputs, production, storage, and transportation have long been influenced by energy prices, the rapid growth of the biofuels sector has created new types of relationships between agricultural and energy markets [5]. Moreover, in periods of financial crises, the volatility of the agricultural market has been very high [6,7]. Those fluctuations on commodity futures markets [8] contribute to the agricultural price oscillations for the farmers. However, not all agricultural products are affected by the same factors, the agricultural market is very diverse in terms of product attributes and particularities which are subject to external factors. For instance, corn futures prices may be determined by certain factors (such as location, transportation costs, contamination, warehouses, weather conditions, etc.) in comparison to wheat prices, that are less sensitive to weather. The changes in climate are something that has been more accentuated in recent years due to the greenhouse effect [9]. Analysis of greenhouse emissions as well as limited resource expenditures or energy consumption is becoming more widespread in precision agriculture (PA) [10] and smart agriculture (SA) [11].

PA is a term that has been coined in recent years. It refers to the concept of using new technologies to increase the yield and profitability of crops while reducing the resources needed for cultivation [12,13]. Thanks to technological advances, farms in developed and developing countries can benefit from the application of low-cost technologies. In this regard, the Internet of Things (IoT) and, more specifically, the Industrial Internet of Things (IIoT), is presented as a key enabling technology for implementing and monitoring resource management solutions in various scenarios in Industry 4.0, including smart agriculture environments [11]. The monitoring of all these values can be carried out with sensors, using new paradigms such as edge computing which enable monitoring while reducing the cost of data analysis in the cloud [14,15] increase the efficiency of agricultural processes [16]. The application of multi-agent systems to monitor agricultural processes is common, especially to achieve the efficient use of land in terms of investment and production [17], to manage resources [18], to increase the efficiency of irrigation systems [19,20], to optimize energy use [21], or predict the prices of agricultural products [22]. Moreover, in the field of selecting suppliers, Valluri and Croson [23] conducted a research towards best supply selection through a game theory approach with agents, in a scenario where reward and punishment were complicated by incomplete information. Over the last years, the number of processes oriented to sustainability objectives [24] and to developed tools [25] is growing. For instance, it has become more popular to integrate environmental, economic, and social attributes when selecting a supplier and sourcing process [26,27,28]. There are many differences in price setting in terms of requirements and the most important attributes for which each model can be built. Most models rely on historical data; however, others focus on the buyer, in which there are sales quotas, decision history polynomials, probabilities, and regressions of the potential prices that might be accepted [29]. In the literature, other approaches have been found, such as agent-based modeling with reinforcement learning conditioned by inventory [30,31]. When product pricing strategies are connected to estimations of price allocation and real-time reporting is taken into account, the relationship between data and price allocation parameters can be modeled dynamically, as demonstrated in the multi-agent supply chain [32]. In the context of a market, goods are exchanged and there are two main players. One of the basic rules in a price system is that goods can be exchanged according to the relative prices of the goods in question. Twenty years ago, Wellman and Wurman [33] already developed the first market-based multi-agent paradigm at a theoretical level.

The above examples demonstrate that multi-agent systems are applied in cases where a single agent is not capable of carrying out all the processes, as several agents and objectives interact in real-time [34]. Although currently there are different MAS focused on price forecast [35] or choice of suppliers [26,27,28], to this day, there is still no MAS in which a buyer can have information on the sustainability of a supplier or a product by monitoring the greenhouse emissions involved in production, the use of pesticides or the consumption of natural resources and having access to information on prices on the futures market. Considering that these needs cannot be fulfilled with a single agent, a MAS has been built [36]. The proposed system consists of MAS that helps choose a supplier through decision-making based on certain attributes. These attributes measure the sustainability of a supplier and also have prices of agricultural futures markets. The different attributes that have been considered are agri-environmental indicators (AEIs) [37]. These parameters consist of water consumption, greenhouse emissions, energy consumption, as well as the use of edge computing technologies that represent an improvement in terms of data transfer costs to the cloud. More concisely, the MAS consists of a preference-based multi-objective optimization problem that is open to real implementation, and therefore each user can fix the desired threshold and input requirements. Although not all products are comparable at the world level, the developed MAS is intended to give an indication of the most recent price developments. Futures markets are an important source of price information for farmers, but only a small percentage of farmers directly trade futures. The availability of high-frequency (intraday) data can help market participants make quicker decisions compared to low-frequency data, such as daily or monthly data. Having high-frequency data allows to better forecast the stock prices [38] so that farmers can sell according to trends in agricultural futures market real prices without having to wait to learn of the effects of selling products.

The rest of this paper is structured as follows. Section 2 consists of a revision of the state of the art of technologies involved in the system. Section 2.1 introduces the edge computing technologies as they play a significant role in monitoring and cost reduction in agriculture and identifying the most important trends in the application of those paradigms in smart farming scenarios. Then, in Section 2.2, state-of-the-art MAS are described, specifically those designated for agriculture scenarios, and finally, in Section 2.3 the deep Q-learning concept is introduced. Section 3 describes the MAS architecture and the data that have been used to conduct the experiment. Section 4 describes the experiments that have been conducted and the results. Finally, Section 5 discusses the solution, implementation fields, conclusions, and future work.

## 2. Related Work

This section reviews the state-of-the-art of the three main topics that are directly related to the case study: the edge computing paradigm, the MAS, and the deep Q-learning algorithm. First, the state-of-the-art of edge computing (EC) paradigm is presented. EC is a paradigm that enables reducing data transmission costs to the cloud, and in this case when conducting the analysis of consumption and greenhouse emissions. Second, the MAS and the multi-objective optimization processes are described to contextualize the conducted experiment in which different agents interact to identify the best supplier and price according to a given preference, and finally an introduction to Deep Q-learning which is the technique used for forecasting the agricultural future market prices.

### 2.1. Edge Computing

The increasing demand for food in terms of quality and quantity has increased the need for industrialization and intensification in the agricultural field [39]. Internet of things (IoT) is a very promising technology that offers many innovative solutions to modernize the agricultural sector [40]. IoT can be used in combination with other technologies such as cloud computing, big data, AI, or distributed ledger technologies (e.g., blockchain) to implement solutions that improve the traceability and productivity of industrial processes [41]. However, when trying to transmit data to the cloud, several challenges arise regarding the privacy of the data, power consumption, or costs associated with the use of cloud services [15]. In this regard, service providers charge fees according to the amount of data that is transferred, stored, and processed in the cloud [42]. By using EC technologies, it is possible to reduce the traffic between the IoT layer and the cloud [14]. EC allows for the execution of machine learning models at the edge of the network, reducing the response time and providing a certain level of service even if the communication with the cloud is interrupted. This is commonplace in scenarios where Internet connectivity is limited (for example, rural agricultural environments) [15]. The EC paradigm has been also used in different studies in which results show that including the costs of edge and non-edge data transfer has an impact on the efficiency [16].

### 2.2. Multi-Agent Systems

Preference-based multi-objective optimization has had an increasing interest in research and academia in the last years [43]. Agents can be defined as intelligent entities with social skills (communication, collaboration, interaction, negotiation, intelligence, coordination, competence) that encapsulate a functionality to solve a problem [34,44]. When two or more agents are able to work together in order to solve a common problem, they form a MAS [34]. MAS are systems that integrate a set of agents that interact, communicate, and coordinate to achieve the established objectives [45]. MAS are designed to meet a set of objectives according to a set of rules and standards. The different designs of MAS have different nomenclatures depending on the methodology, nevertheless, they tend to include social, communicative, interactive, and normative aspects [46]. Each of these is described below.

1 Social aspects refer to the description of the set of roles, groups (role associations), and the relationship between them. Regarding the existing relationships between roles and groups (recursively), some authors have defined a set of social structures that allow to model the interactions between members. Among the main structures, the following stand out: hierarchies, coalitions, teams, congregations, societies, federations, markets, matrices, and composite organizations. Some studies have simply defined possible relationships between members [46] such as dependency, hierarchy, use, etc.
2 The communication aspects refer to the means that makes the exchange of information possible. That is, a knowledge representation language (usually represented by an ontology) and a communication language. The communication sequence between two agents is called illocution [47], communication act [46], or link [48].
3 Interaction aspects refer to how roles collaborate to achieve common goals. There might be objectives that cannot be achieved individually, and that require the combination of several agents for achievement, and it is necessary to describe an interaction structure that allows to articulate or regulate the achievement of individual sub-objectives that in turn make the achievement of higher-level objectives possible [46].
4 Normative aspects: note that this is one of the main pillars of organizational MAS [49]. Norms (or institutional patterns make it possible to establish a relationship of trust between the members of an organization, as they limit the free will of individual agents [50].

In addition to the concepts that have just been presented (role, organization, norms, and social structures), organizational MAS routinely include another key concept: Environment. Agent theory traditionally conceives the agent as an entity that plans its actions on the basis of its perception of the environment. However, the increasing complexity of the environment itself in the context of open systems (dynamic, heterogeneous, and unpredictable) can not only make the MAS unpredictable, but also difficult to interact with [34,44].

Moreover, it is important to understand the effect of ubiquitous automated agents on the performance of economic systems. With a special emphasis on being able to achieve, at the computational level, the capacity for some agents to reason about the reasoning of other agents and of humans who would also be at stake. Moreover, these agents will adopt a game theory vision [51], where each agent will act according to the behaviour of the other agent (in the best and most rational manner for both agents). Game theory consists of a mathematical theory that studies interactions among self-interested agents [52]. The traditional game theory was revised and applied in biology by the authors of [53], in which the authors determined the concept of Evolutionary Stable Strategies (ESS). In these strategies, there were not only two players in a complete information situation and that was a condition of Nash’s equilibrium [54]. Therefore, it would lead to equilibrium situations that are part of the traditional economy. The quality of an AI design is determined through the degree to which the agent’s actions achieve specific objectives, subject to observed perceptions. If we express objectives in terms of preference over results and perceive both perception and action within the framework of decision-making under uncertainties, then the position of the AI agent is fully in line with the standard economic paradigm of rational choice. Consequently, the task of the AI developers is to build rational agents, or agents that are as rational as possible, given the limits of their computational resources [55]. At the multi-agent level, a developer cannot directly program the behaviour of AI, but instead determines the rules and incentives that will regulate the interactions between AI. The authors of [56] propose a multi-agent system to simulate group decision-making processes, where agents are designed with emotional properties and reason using incomplete information.

In real-world scenarios, there are multiple applications and situations within a given market, different agents have to make decisions with incomplete information. Methods such as the game theory for portfolio optimization can be used in these cases, regardless of the product in question [57]. The application of computing techniques for the optimal product or supplier portfolio, from the application of machine learning algorithms [58], to genetic algorithms for product optimization [59], neural networks [60], deep neural networks [61], and reinforcement learning [62].

#### Multi-Objective Optimization Problem

A multi-objective optimization problem can be defined as the following Equation (1), where f(x) is the *k*-dimensional objective vector.

(1) $$\mathrm{Max}f\left(x\right)=(f_{1}\left(x\right),f_{2}\left(x\right),\ldots f_{k}\left(x\right))$$

The multi-attribute utility function is used to represent the preferences of a user over packages of goods, under conditions of certainty about the results of any potential choice. Van Calker et al. [63] presented a model of the sustainability multi-attribute function for evaluating sustainability in different farming systems.

Preferences can be characterized by utility functions, where the information regarding preference is implicitly involved in the function, enabling the ranking of solutions. Utility functions assign different weights to given attributes. The utility function for the buyer agent in this case would be the following, in which product *A* (which could be Corn sold at *X* price and Greenhouse contamination of *Y* points), is preferred over product *B* only if the expectation of the function *U* is higher under *A* than under *B*, as shown in Equation (2).

(2) $$E_{A}\left[u\left(x_{1},\ldots ,x_{n}\right)\right]>E_{B}\left[u\left(x_{1},\ldots ,x_{n}\right)\right]$$

### 2.3. Deep Q-Learning Algorithm

Reinforcement learning (RL) consists in an agent interacting with the environment, learning an optimal policy, by trial and error, for sequential decision-making problems [64]. The standard RL consists of an agent interacting with an environment, which can be modeled as a Markov decision process (MDP).

The Q-learning algorithm [65] is one of the best-known, model-free techniques in RL and has numerous evolutions and variants [66]. Q-learning, is a model-free off-policy RL method, which consists of agents whose objective is to reach the state-action-value of a function Q=(s,a) by interacting in a given environment. As the agent explores the environment, *Q* returns an increasingly accurate approximation of the expected value of an action *a*, given a state *s* of the expected value of an action *a*, given a state *s*. That is, the function *Q* is progressively updated. Q-learning [67] can be defined as a way for agents to learn how to act optimally in controlled markovian domains, which means the future depends only on the current state and action, but not on the past. It is formulated as an MDP which can be defined by the 5-tuple (s,a,p,r,γ), where *s* is the state, *a* is the action, *p* is the transition probability, *r* is the reward function, and γ is the discount factor.

Deep learning (DL) has accelerated progress in RL, with the use of deep learning algorithms within RL defining the field of deep reinforcement learning (DRL) [66]. DL allows RL to be extended to previously intractable decision-making problems, i.e., environments with a high number of dimensional states and action spaces. As a neural network is a universal functional approximation, it can be used as a substitute for the Q-table. In the learning process, DL optimizes the weights, θ, to minimize the error estimated by the loss function. The error or loss is measured as the difference between the predicted result and the actual result. The deep Q-network (DQN) was first introduced by [68] and then [69] introduced additional techniques, such as DQL. The base algorithm for DQN is value-based RL, which is a method that approximates an action value (i.e., a Q-value) in each state. An algorithm based on Q-learning that approximates the Q-function using DNN is the basis of DQN [69]. To prevent DNN from learning only through the experience of a specific situation, experience replay has been introduced to sample a general experience batch from memory [69]. In reinforcement learning, the temporal difference (TD) target function is always unknown. Before an agent takes an action, the Q-value can be defined as Q(s,a), and after the action is taken the new state is $R\left(s,a\right)+\gamma max_{a^{\prime }}Q\left(s^{\prime },a^{\prime }\right)$, so the temporal difference is defined in Equation (3).

(3) $$T\left(a,s\right)=R\left(s,a\right)+\gamma \times max_{a^{\prime }}Q\left(s^{\prime },a^{\prime }\right)-Q_{t-1}\left(s,a\right)$$

The value function is approximated by a neural network Q(s,a;θ) with a parameter, θ where the parameter is learned by minimizing the TD loss. Thus, the loss function turns out to be

(4) $$Loss=Q^{*}\left(s_{t},a_{t}\right)-Q\left(s_{t},a_{t}\right)$$

The key idea of DQN is to learn an approximation of the optimal value function *Q*, which conforms to the Bellman optimality equation [70]. In the DQN algorithm, the *Q* corresponds to the function that represents the expected rewards for a given action in a given state. DQN refines the policy with respect to action values by the max operator [71]. One way to minimize the loss function is by the gradient descent method [72]. In this method, the policy Q(s,a) is updated on the basis of the current reward and the maximum value of the expected future rewards. In the DQN, the learn function can be described as in Algorithm 1, where ϵ is the learning rate and π is the optimal policy.
**Algorithm 1:**Algorithm adapted from the work in [71].
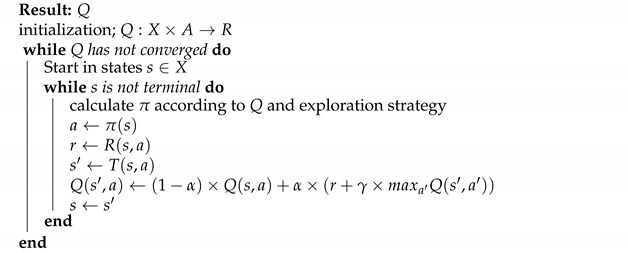


## 3. Case Study

This section describes the MAS that has been designed and how it has been conducted. This section has two main parts: first, the architecture of the MAS is defined in Section 3.1, and second, the different types of data used to perform the experiment are described in Section 3.2.

### 3.1. Architecture

**Figure 1:**
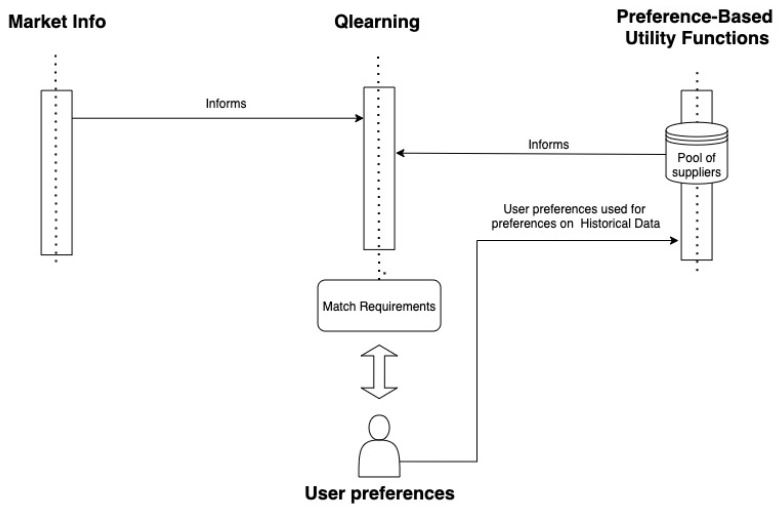
Proposed multi-agent system.

The architecture that has been designed for this case study is represented in Figure 1. A MAS architecture has been used as a MAS can be defined as a collection of, possibly heterogeneous, computational entities, having their own problem-solving capabilities and which are able to interact in order to reach an overall goal. In the case of this investigation, each agent has a role [73]. As shown in Figure 1, there are three agents that communicate to provide the best option according to user preferences; it is a closely collaborating agent system in which every agent has its own specialized capabilities and knowledge, and no single agent has full knowledge of the world. The main functions include agricultural futures market information retrieval, deep Q-learning for lowest price setting, and preference based weight system for users’ preferences. The architecture designed for the MAS has been implemented with SPADE library [74].

In this case, to test the MAS, one agent collects information from the period described in Section 3.2. Then, the preference-based utility functions retrieve information from the historical database and also the preferences of the user in terms of weights for each attribute. In the case of this study, as the approach is preference-oriented and there are some weights according to each attribute, the Equation is represented as in Equation (5). For the values of the other attributes, the equivalence is shown in Equation (6).

(5) $$\left(w_{2},\cdots ,w_{n}\right)\left(w_{2},\cdots ,w_{n}\right)$$

(6) $$\left(y_{1},w\right)∼<\left(x_{1},w\right):\left(z_{1},w\right)>$$

Next, the deep Q-learning agent is responsible for modeling nonlinear trends of stock price time series, by predicting values and identifying the lowest prices.

### 3.2. Data

**Figure 2:**
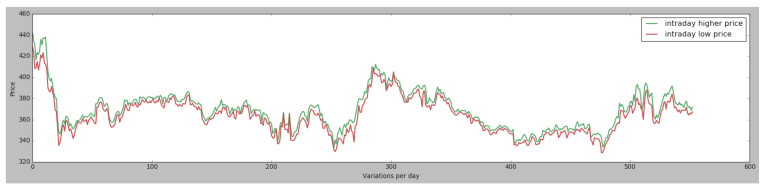
CZ symbol price variation.

The data that have been chosen for testing the MAS can be modified and adapted to other products or markets. To implement the case study, information has been taken from a specific time-period, but as mentioned above the presented architecture is adaptable to any market. For the development of the model, two main datasets have been used; on the one hand real data from agricultural futures market and on the other hand a synthetic dataset for potential suppliers. The synthetic dataset has been built with different agri-environmental indicators (AEIs) [37] as main attributes for each supplier. AEIs track the integration of environmental concerns in the common agricultural policy (CAP) at EU, national and regional levels. The different attributes that have been considered are in Table 1 as follows:

In the case of the qualitative values, such as the edge computing attribute, the values were converted into the following values [0,1]. The other data that are considered in the study are the data from the agricultural futures market. To evaluate the model and to see how it works, information has been collected from the corn agricultural futures market, which has the CZ symbol. The period for which the data has been collected is from 2017 till 2019, because the 2020 and 2021 values are very irregular due to the COVID-19 pandemic. The attributes that are usually collected from the stock market are the ones defined in Table 2; nevertheless, the most commonly used inputs for next-day stock price prediction in the literature are the stock index opening or closing prices [75], and those are the values have been gathered for the case study, as represented in Figure 2.

## 4. Results

The problem that the presented MAS overcomes is the difficulty of choosing a supplier according to sustainability parameters, as well as having transparent market information. The MAS presented helps potential buyers to identify a supplier according to their preferences in terms of sustainability as well as efficient use of resources. It also combines the selection of the supplier according to certain preferences, as well as seeing the actual price differences with the quotations of the products and being able to buy at the most optimal times. The designed MAS has two main results that are presented below. The first result is related to the supplier selection ranking, and the second one to price forecasting.

To achieve the first goal, different values have been assigned to the attributes, in terms of user preferences. To test the model, different cases have been tested with the following preferences as represented in Table 3.

**Figure 3:**
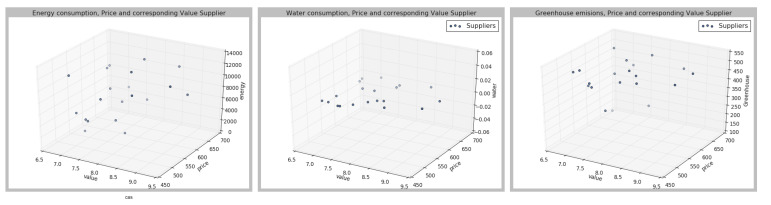
Case 1, according to the preferences described in Table 3.

**Figure 4:**
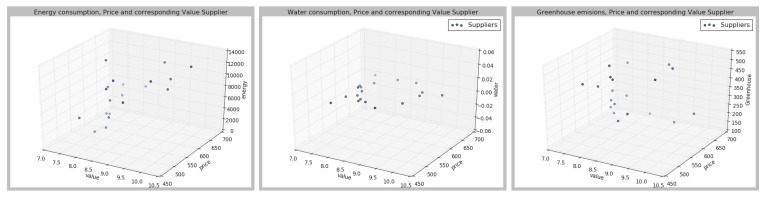
Case 2, according to the preferences described in Table 3.

**Figure 5:**
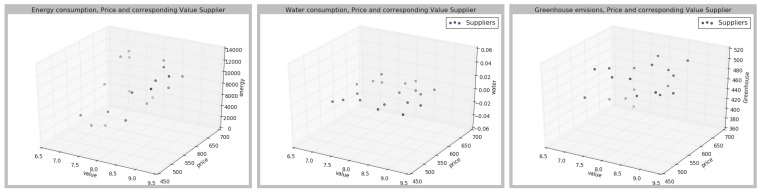
Case 3, according to the preferences described in Table 3.

Then, a value between [0,10] is assigned to each supplier, where value is understood as the total sum of the weights of different attributes, assigned according to the attribute, and attribute importance. The outputs and the values of each of the parameters are represented in the Figure 3, Figure 4 and Figure 5, in which three dimensions are represented; the value according to the different weights for each of the parameters.

As can be seen from Figure 3, Figure 4 and Figure 5, the different suppliers’ selection would vary according to the preferences set by the user. Once the different suppliers have been ranked, the results are sent to the Q-learning agent. The deep Q-learning agent’s goal is to maximize the total amount of reward it receives. In this case, the agent’s goal is to buy at the lowest price given a certain amount of money. Therefore, the user should fix an initial amount of money, which in this case has been 20,000. To build the agent, the different parameters have been fixed as shown in Table 4. Then, different hyperparameters have been changed to identify the best agent performance. The set hyperparameters are shown in Table 4, and then the other hyperparameters have been compared, as represented in Table 5.

**Figure 6:**
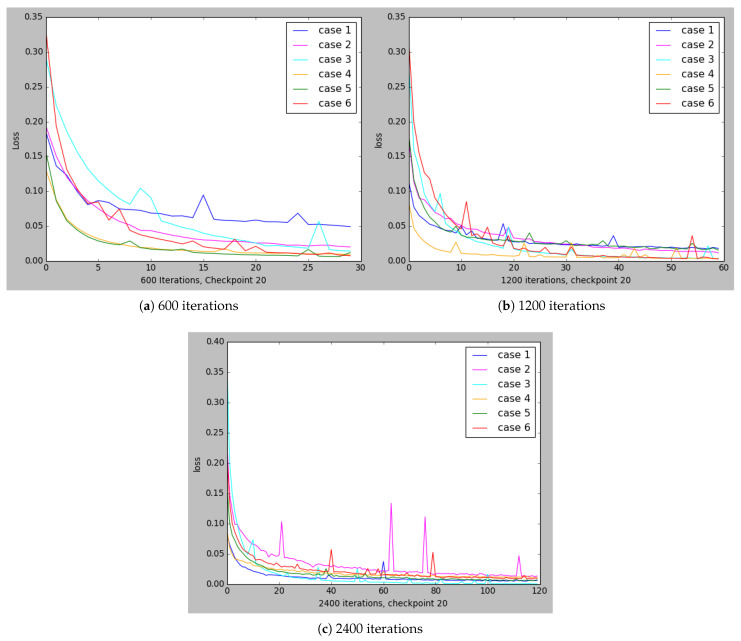
Loss function comparison on cases from Table 5 with different iterations.

The Q-learning agent has one hidden layer with 256 neurons and the activation function is the rectified linear unit (ReLU) [76]. In the learning process, DL optimizes the weights, θ, to minimize the error estimated by the loss function. Therefore, the error or loss are measured as the difference between the predicted result and the actual result. The loss function for the different cases which are described in Table 5, is represented in Figure 6.

**Figure 7:**
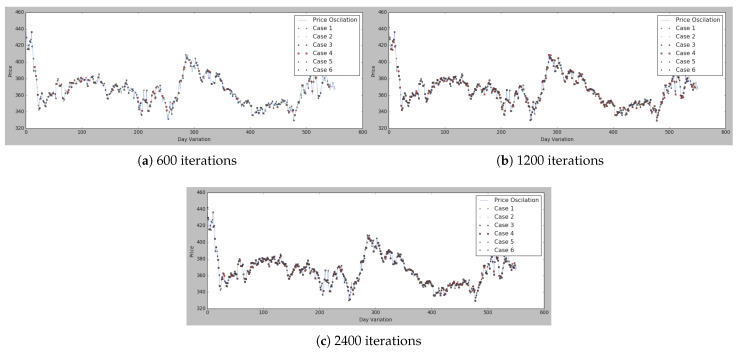
Lowest price identification with different iterations on the basis of the parameters described in Table 5 for each case.

**Figure 8:**
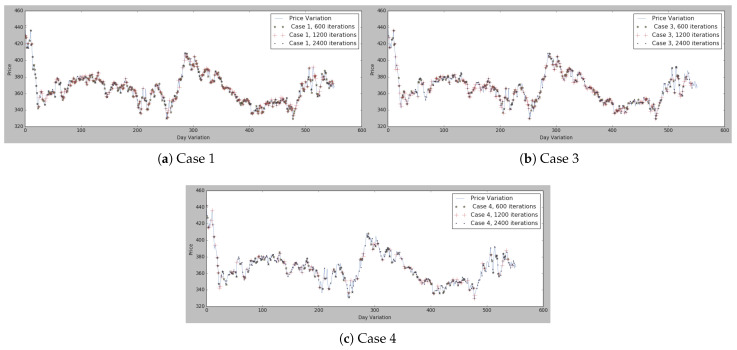
Table 5 for three selected cases.

As can be seen in Figure 6, cases 3 and 4 are the ones achieving the best results in all Figure 6a–c, while, for instance, case 1 has the worst results in Figure 6a but then has better results in Figure 6b. Moreover, in Figure 7 and Figure 8, all the marked values are placed in the real price variation line.

To view the comparisons of cases 1, 3, and 4 in more detail, the results are represented in Figure 7, where all the different cases are compared and represented according to the number of iterations in Figure 7a–c. Has been collected the different cases that were performing better according to the loss functions of Figure 6.

The conclusion that can be drawn from Figure 6 and Figure 7 is that the best options can be chosen by basing the decision-making on hyperparameters, either case 1, 2, or 3, so this case’s comparisons have been compared in Figure 8. As can be seen in Figure 8a, case 1 presents good results either in case of 1200 iterations and 2400. Therefore, the option that included in the MAS is the one with the above-mentioned hyperparameters. Thus, the buyer is at last presented with a potential list of suppliers and the lowest market prices at a given moment, which allows them to identify the most suitable suppliers for them as well as the best times to buy the products and also to identify the trends in specific products.

## 5. Discussion

The use of new technologies as well as the reduction of natural resource consumption or the generation of greenhouse effect impacts is becoming a more important element when choosing a supplier [77]. In the agricultural market, wholesalers purchase and store products in a well-controlled environment in the harvest season, and then recover selected quantities to sell in the market. The amount that is purchased in the harvest season, as well as the amount that is recovered in each selling period, have a strong impact on a wholesaler’s profit [78]. The proposed MAS allow to buy agricultural products sustainably thanks to the use of technologies such as edge computing, which reduces agricultural costs and help to make efficient use of resources [16] such as water [20,79] or energy optimization [21], as well as monitoring harmful emissions. According to the work in [78], an optimal selling policy can increase the expected profit. Therefore, being able to combine and obtain real-time price information allows adjusting purchase prices in anticipation of the problem of making strategic sales decisions [80]. In the current case, a paradigm in which each stakeholder (i.e., direct FMCG chains, wholesalers, etc.) acts individually is proposed. For a further evaluation of the model, a pool of suppliers will be contacted in order to include location as another interesting factor when determining prices and creating a reality between the requirements of potential buyers and suppliers.

## Figures and Tables

**Table 1:** Supplier attributes.

Attribute	Description	Measure Units
Energy consumption	Energy consumed	kWh
Greenhouse	$N_{2}O$ is produced mostly from excess nitrogen in soils; one way to suppress emissions of this gas is to apply fertilizer judiciously: adding just enough, at the right place and time, to meet crop demands, but avoiding excess amounts. This can reduce fertilizer costs for producers and reduce the amount of nitrogen lost through excess fertilizer application	$N_{2}O$
Water consumption	Amount of water consumed per year for the irrigation of crops	mL/ha
Edge Computing Techniques	Whether edge computer techniques are used to reduce the cost of using the cloud and make their sensors communications more robust and scalable with the cloud	Boolean (Yes/No)

**Table 2:** Stock market price variation. Source: Stock market for the symbol CZ.

Attribute	Description
Date	The information regarding price variations is considered per day
Price	Stock price
Open	Stock price at the opening
High	Highest price within a concrete day
Low	Lowest price within a concrete day
Vol.	Number of stocks
Change %	Variation regarding previous date

**Table 3:** Case study combinations according to weights assigned [1,10] for the attributes of each supplier.

	Greenhouse	Water	Energy
Case 1	3	5	2
Case 2	0	8	2
Case 3	8	1	3

**Table 4:** Fixed hyperparameters that have been used for the different case combinations in the deep Q-learning agent.

Parameter	Description	Value
γ	Maximizes the current reward	0.950
ϵ	Either taking random actions or using the trained actions	0.500
ϵ decay	The decrease over time in the use of the random and trained actions	0.999
Actions	The actions that can be taken by the agent, which is either selecting a buying price or do nothing	2
Replay memory size	Agent’s experiences at each time step in a data set	1000

**Table 5:** Parameters used for each case comparison related to the different cases.

Case Number	Window Size	Batch Size
1	8	32
2	10	32
3	20	32
4	8	64
5	10	64
6	20	64
